# Dynamic Profile of S-Layer Proteins Controls Surface Properties of Emetic *Bacillus cereus* AH187 Strain

**DOI:** 10.3389/fmicb.2022.937862

**Published:** 2022-06-29

**Authors:** Cécile Boutonnet, Sébastien Lyonnais, Beatrice Alpha-Bazin, Jean Armengaud, Alice Château, Catherine Duport

**Affiliations:** ^1^Avignon Université, INRAE, UMR SQPOV, Avignon, France; ^2^CEMIPAI, UAR3725, CNRS, Université de Montpellier, Montpellier, France; ^3^Département Médicaments et Technologies pour la Santé (DMTS), Université Paris Saclay, CEA, INRAE, Bagnols-sur-Cèze, France

**Keywords:** S-layer proteins, *Bacillus cereus*, surfaceome, surface properties, adhesion

## Abstract

Many prokaryotes are covered by a two-dimensional array of proteinaceous subunits. This surface layers (S-layer) is incompletely characterized for many microorganisms. Here, we studied *Bacillus cereus* AH187. A genome analysis identified two genes encoding the S-layer proteins SL2 and EA1, which we experimentally confirmed to encode the two protein components of the S-layer covering the surface of *B. cereus*. Shotgun proteomics analysis indicated that SL2 is the major component of the *B. cereus* S-layer at the beginning of exponential growth, whereas EA1 becomes more abundant than SL2 during later stages of stationary growth. Microscopy analysis revealed the spatial organization of SL2 and EA1 at the surface of *B. cereus* to depend on their temporal-dynamics during growth. Our results also show that a mutant strain lacking functional SL2 and EA1 proteins has distinct surface properties compared to its parental strain, in terms of stiffness and hydrophilicity during the stationary growth phase. Surface properties, self-aggregation capacity, and bacterial adhesion were observed to correlate. We conclude that the dynamics of SL2 and EA1 expression is a key determinant of the surface properties of *B. cereus* AH187, and that the S-layer could contribute to *B. cereus* survival in starvation conditions.

## Introduction

Numerous bacteria and almost all archaea are covered by a two-dimensional porous paracrystalline lattice. This 5–70 nm thick lattice is known as the Surface layer (S-layer; Bharat et al., 2021; Pum et al., 2021). The S-layer is composed of one or two extracellular 40-200-kDa (glyco)proteins (Sára and Sleytr, 2000), called S-layer proteins (SLP), that self-assemble into a number of symmetries: oblique (p1 and p2), square (p4), and hexagonal (p3 and p6; Sleytr et al., 1999). Most S-layers cover upwards of 70% of the microorganism’s surface area (Sleytr and Beveridge, 1999). In bacteria, the S-layer is composed of approximately 5 × 10^5^ SLP subunits punctuated by identically-sized pores distributed throughout the 2D network. These pores measure 2 to 8 nm in diameter depending on the individual microorganism (Åvall-Jääskeläinen and Palva, 2005; Sleytr et al., 2014). Although very diverse in terms of sequence length, the primary structures of SLP have a bipartite organization, comprising an amino acid sequence involved in 2D lattice formation, and a sequence that serves to anchor the structure to the cellular envelope. Whereas the lattice-forming sequences have a remarkably divergent composition, the anchoring sequences show little sequence variability (Zhu et al., 2017). In some Gram-positive bacteria, like *Bacillus anthracis*, the anchoring sequences of SLPs contain three tandem cell-wall-binding S-layer homology (SLH) domains (Château et al., 2020; Ravi and Fioravanti, 2021). The SLH domains of the two SLP proteins produced by *B. anthracis* (SAP and EA1) are located at the N-terminus of the protein sequences, and bind non-covalently to peptidoglycan-linked pyruvylated secondary cell wall polymers (SCWPs; Mesnage et al., 1997, 2000; Kern et al., 2011). In addition to SAP and EA1, *B. anthracis* synthesizes and secretes 22 other SLH domain-containing proteins (Kern and Schneewind, 2008; Fagan and Fairweather, 2014). These proteins embed within the S-layer lattice and are thus designated S-layer-associated proteins (SLAPs). These proteins may contribute to the functions assigned to S-layers (Kern et al., 2010, 2012; Anderson et al., 2011).

A multitude of functions have been suggested for prokaryotic S-layers (Fagan and Fairweather, 2014; Zhu et al., 2017). From a general point of view, S-layers play an important role in regulating cell shape, growth, and survival. In their interactions with humans, S-layers are directly involved in bacterial pathogenesis. For example, disruption of S-layer integrity in *B. anthracis* prevents lethality in a mouse model of anthrax (Fioravanti et al., 2019). The S-layer also contributes to the pathogenesis and adaptation of *B. cereus* group species through various mechanisms such as adhesion to extracellular matrices and intestinal cells (Kotiranta et al., 1998; Auger et al., 2009; Sanchez et al., 2009), and intraocular inflammation (Mursalin et al., 2019, 2020). In survival terms, the S-layer also contributes to environmental adaptation, as it protects bacteria from harmful environmental conditions, such as low pH, radiation, high temperatures, osmotic stress and lytic enzymes, antimicrobial peptides, and bacteriophages (Aravindh et al., 2015; Gerbino et al., 2015).

In this study, we investigated the structure, biogenesis, and properties of the S-layer produced by *B. cereus* AH187. This bacterial strain, also known as F4810/72, is a human pathogen causing emetic foodborne illnesses (Ehling-Schulz et al., 2005). Emetic symptoms are caused by cereulide, a dodecadepsipeptide produced by *B. cereus* in contaminated food prior to consumption (Agata et al., 1995, 2002). Like other emetic *B. cereus* strains, *B. cereus* AH187 is phylogenetically close to *B. anthracis* strains (Guinebretière et al., 2008; Carroll et al., 2019), and is covered by an S-layer (Duport et al., 2020; Rousset et al., 2020; Château et al., 2022). With this study, we showed that the S-layer of *B. cereus* AH187 self-assembles from two SLPs: EA1, which is quite similar to its homolog in *B. anthracis*, and SL2, which presents clear differences with respect to *B. anthracis* SAP. Proteomics analyses and microscopy observations revealed SL2 to accumulate at the cell surface from the beginning of growth, whereas the level of EA1, present as patches, reached a maximum during the late stationary growth phase. Deletion of the *sl2* and *eag* genes encoding SL2 and EA1, respectively, changed the surface properties of *B. cereus* AH187 and revealed the importance of the S-layer in stationary *B. cereus* adhesion.

## Materials and Methods

### Bacterial Strains and Growth Conditions

The wild-type strain used in this study was *B. cereus* AH187 (F4810/72), which was originally isolated from vomit (Turnbull et al., 1979). The *Δsl2Δeag* double mutant was constructed by allelic exchange (Arnaud et al., 2004), applying the previously reported experimental procedure (Château et al., 2022). The pMAD plasmid to achieve allelic exchange was created as follows: the 1-kbp DNA sequences flanking the 5’end of BCAH187_A1064 (up-*sl2*) were amplified by PCR using primer pairs upF-sl2-SalI and upR-sl2-Sac-NheI-SacII; and the 3’end of BCAH187_A1065 *(down-eag)* was amplified by PCR using the primer pairs downF-eag-NheI-SacII and downR-*eag*-*Nco*I (Supplementary Table S1). Both amplicons were cloned independently into pCR 2.1-TOPO (Invitrogen). The *down-eag* DNA fragment was excised from pCR 2.1.-TOPO by digestion with *Sac*I-*Sac*II and inserted between the *Sac*I-*Sac*II sites of the pCR 2.1-TOPO plasmid containing up-*sl2*. An *Nhe*I-*Sac*II fragment containing a spectinomycin resistance cassette (Guérout-Fleury et al., 1995) was then inserted between the up-*sl2* and down-*eag* fragments. The resulting up-*sl2*::*spec*::down-*eag* DNA fragment was then isolated using *Nco*I and *Sal*I and cloned into pMAD digested with the same enzymes. The resulting pMAD-up-*sl2-spec-*down-*eag* plasmid was used to create the *Δsl2Δeag* mutant. Replacement of *sl2* and *eag* by the spectinomycin resistance cassette was verified by PCR with appropriate oligonucleotide primers (Supplementary Table S1). Complementation experiments could not be performed, as attempts to clone the large *sl2*-*eag* DNA fragment into pHT304 failed.

Wild-type (WT) *B. cereus* AH187 and the *Δsl2Δeag* mutant strains were routinely grown in Lysogeny broth (LB) medium. For proteomic, microscopic, and phenotypic analyses, bacteria were aerobically cultivated in chemically-defined MOD medium (Rosenfeld et al., 2005) supplemented with 30 mM glucose (MODG) at 37°C.

### Heterologous Expression of *Bacillus cereus* SL2, and Anti-SL2 Antibody Production

The *B. cereus* BCAH187_A1064 ORF (*sl2*), without its signal peptide, was amplified using specific primers (Supplementary Table S1) in preparation for cloning into the expression plasmid pET100 directional TOPO (Invitrogen). The construction was verified by sequencing, and the recombinant plasmid was used to transform *Escherichia coli* BL21 Star (DE3; Invitrogen). Recombinant cells were grown on LB at 37°C to an OD_600_ of 0.6–0.8. Isopropyl β-D-thiogalactopyranoside (IPTG) was then added (final concentration 1 mM). After 3 h at 30°C, cells were harvested by centrifugation (7,000 g, 15 min, 4°C). The pellet was resuspended in equilibration buffer (20 mM Na_2_PO_4_, 300 mM NaCl, 10 mM imidazole), and cells were disrupted using a FastPrep homogenizer, applying three 45-s pulses at 6 m/s (MP biomedicals). Samples were centrifuged (7,000 g, 10 min, 4°C), supernatant was collected and incubated for 30 min at room temperature (RT) with 700 μl of functionalized HisPur Ni-NTA beads (Invitrogen). Beads were washed twice with washing buffer (25 mM imidazole in PBS) before eluting recombinant SL2 with 300 mM imidazole in phosphate buffer (PBS). An aliquot of SL2 eluate (1 mg) was run on a 10% SDS-polyacrylamide gel. The SL2-containing band was excised from the gel and used to produce anti-SL2 polyclonal antibodies in rabbits (Eurogentec, Seraing, Belgium). The specificity of the antibodies produced was tested by western blotting. Blot images were analyzed using a Molecular Imager ® ChemiDoc™ XRS+ (Bio-Rad), and images were processed with Image Lab™ software.

### Immunofluorescence Microscopy

WT cells were grown in MODG medium and collected by centrifugation (7,000 g, 5 min, 4°C) at early exponential (EE, OD_600_ = 0.3), late exponential (LE, OD_600_ = 2.5), stationary (S, OD_600_ = 3.5), and late stationary (TS, OD_600_ = 4.2) growth phases. Cells were fixed overnight with 4% paraformaldehyde at 4°C, washed twice with PBS, and then blocked with 2% Bovine Serum Albumin (BSA) for 30 min at RT under shaking. Cells were incubated with anti-EA1 (Wang et al., 2014) and anti-SL2 primary antibodies (diluted 1:500 and 1:200, respectively) for 1 h at RT, washed twice with PBS and then incubated with Alexa Fluor 594-conjugated goat anti-rabbit polyclonal antibodies (diluted 1:1000, ThermoFisher, A-11012). Images were captured using an Olympus BX61 microscope. Fluorescence microscopy images were analyzed using Fiji and Microbe J software (Ducret et al., 2016). Experiments were performed in triplicate.

### Extraction of Extracellular Non-covalently Bound Cell Surface Proteins and Immunodetection of SLPs

Non-covalently bound cell surface proteins that include SLPs and SLAPs were extracted from WT and *Δsl2Δeag* mutant cells at early exponential (EE, OD_600_ (WT) = 0.3 ± 0.0 and OD_600_ (*Δsl2Δeag*) = 0.24 ± 0.1), late exponential (LE, OD_600_ (WT) = 2.6 ± 0.3 and OD_600_ (*Δsl2Δeag*) = 2.7 ± 0.2), stationary (S, OD_600_ (WT) = 4.1 ± 0.5 and OD_600_ (*Δsl2Δeag*) = 3.5 ± 0.5), late stationary 1 (TS1, OD_600_ (WT) = 6.8 ± 1.7 and OD_600_ (*Δsl2Δeag*) = 7.4 ± 2.1), and late stationary 2 (TS2, OD_600_ (WT) = 8.1 ± 1.9, and OD_600_ (*Δsl2Δeag*) = 11.4 ± 3.6) growth phases, using 3 M urea buffer, as previously described (Château et al., 2022). Proteins were quantified using the BCA assay kit (Pierce). SLPs were detected by western blotting with rabbit anti-SL2 (1:10,000) or anti-EA1 (1:10000) antibodies applied for 2 h at RT. Immunoreactive products were revealed after incubation with Horseradish Peroxidase (HRP)-conjugated-anti-rabbit antibodies and ECL staining. Images were analyzed as described above.

### Peptide Fractionation, Mass Spectrometry, and Analysis

WT and ∆*sl2*∆*eag* cells were grown in MODG medium and collected by centrifugation (7,000 g, 5 min, 4°C) at EE, LE, S, TS1, and TS2 growth phases. Non-covalently bound cell surface proteins from a total of 30 samples (three biological replicates × five time-points × two strains) of WT and *Δsl2Δeag* were subjected to a short electrophoretic migration on NuPAGE 4–12% Bis-Tris gels (Invitrogen), using NuPAGE MES supplemented with NuPAGE antioxidant as running buffer (Hartmann and Armengaud, 2014). Proteins were proteolyzed *in gel* with sequencing-grade trypsin (Roche) according to the ProteaseMAX protocol (Promega). Samples were submitted to Liquid Chromatography with Tandem Mass spectrometry (nanoLC-MS/MS) on a Q-Exactive HF mass spectrometer coupled to an Ultimate 3000 nano LC system (Thermo Fisher Scientific, Illkirch-Graffenstaden, France). NanoLC-MS/MS analysis was performed as follows: peptides were loaded for online desalting on a reverse-phase Acclaim PepMap 100 C18 precolumn (100 Å pore size, 300 μm i.d. × 5 mm), and then resolved for 90 min on a nanoscale Acclaim PepMap 100 C18 column (3-μm bead size, 100-Å pore size, 75 μm id × 500 mm) at a flow rate of 200 nl.min^−1^.Tryptic MS/MS spectra were searched against the *B. cereus* AH187 NCBI_20200622 database using the MASCOT Daemon search engine with the following parameters: 5 ppm peptide mass tolerance, 0.02 Da MS/MS fragment mass tolerance, 2^+^ or 3^+^ peptide charge, a maximum of two missed cleavages, cysteine carbamidomethylation (+57.0215) as fixed modification, and Met oxidation (+15.5949) as variable modification. Only peptides identified at value of *p* ≤ 0.05 in homology threshold mode, and proteins identified by at least two distinct peptides were retained upon parsing with IRMa software v1.3.1, as recommended (Christie-Oleza et al., 2012). The false discovery rate determined from the corresponding decoy database was estimated to be less than 1%. Spectral counts, defined as the number of MS/MS spectra assigned per protein, were determined for all validated proteins as previously described (Cogne et al., 2019). Protein abundance was compared between WT and *∆sl2∆eag* mutant strains by applying the TFold test (Carvalho et al., 2012). Proteins were considered to be differentially accumulated when fold-change ≥1.5 and *p* ≤ 0.05. The mass spectrometry proteomics data have been submitted to the ProteomeXchange Consortium *via* the PRIDE partner repository under dataset identifiers PXD033486 and 10.6019/PXD033486 for the surface proteome of WT *B. cereus* AH187, and PXD033494 and 10.6019/PXD033494 for the surface proteome of the ∆*sl2∆*eag mutant.

### Transmission Electron Microscopy Analysis

WT and ∆*sl2*∆*eag* cells grown overnight in MODG medium were collected by centrifugation (7,000 g, 5 min, 4°C), and washed twice with 10 mM Tris–HCl pH 8. Bacteria pellets were then resuspended in 25 mM Tris–HCl containing 10 mM MgCl_2_, and vortexed three times for 30 s with glass beads (0.01 mm). Cells were fixed with 0.25% glutaraldehyde. Fixed cells (20–30 μl) were deposited on 400 mesh copper grids covered with a formvar membrane (Delta, Mauressac, French microscopies), dried by blotting with filter paper, before applying three drops of the anionic negative staining agent ammonium molybdate (1% (w/v)) for 10–20 s. Cells were observed using a HT7800 Hitachi transmission electron microscope (Hitachi, Tokyo, Japan) at an acceleration of 80 kV. Electron micrographs were recorded using an XR401, sCMOS (AMT, Woburn, MA-US) AMT camera.

### Atomic Force Microscopy Analysis

For Atomic force microscopy (AFM) measurements, FluoroDish cell culture dishes (World Precision Instruments, United Kingdom) were coated overnight at 4°C with 0.1% poly-L-lysine (Sigma), washed with PBS, air dried and stored at 4°C. AFM imaging was performed at RT on a NanoWizard IV atomic force microscope (JPK BioAFM, Bruker Nano GmbH, Berlin, Germany) mounted on an inverted microscope (Nikon Ti-U, Nikon Instruments Europe B.V, Amsterdam, Netherlands), and equipped with a standard monochrome CCD camera (ProgRes MFCool, Jenoptik, Jena, Germany). A software module (DirectOverlay, JPK BioAFM, Bruker Nano GmbH, Berlin, Germany) was used to calibrate the tip position with the optical image. Bacteria were detected in bright field with a 100× objective (Nikon CFI Apo VC, 1.4 NA, oil immersion). AFM topographic images were obtained using the quantitative imaging (QI) mode. Before each set of acquisitions, the sensitivity and spring constants of the cantilever were calibrated (thermal noise method). Using the JPK SPM-data processing software, images were flattened by fitting to a polynomial/histogram line. Low-pass Gaussian and/or median filtering was subsequently applied to remove minor noise from the images. Particle height, based on the height (measured) channel of the QI mode, was analyzed using the cross-section tool in the analysis software. For height and adhesion measurements, the WT and ∆*sl2*∆*eag* strains were grown to EE phase [OD_600_ (WT, ∆*sl2*∆*eag*) = 0.4 ± 0.0] in 10 ml of MODG, then collected and washed three times in PBS by gentle centrifugation (4,500 g, 10 min at 4°C) to maintain an intact surface as far as possible. The pellet was fixed for 2 h in 1 ml of fixation buffer (2.5% glutaraldehyde with 0.1 M cacodylate, pH 7.1) before washing and storing in PBS at 4°C. Sample concentrations were adjusted to OD_600_ = 0.1 before depositing the suspension on the functionalized Petri dishes. Images were recorded with an MLCT bio-D cantilever (mean cantilever spring constant = 0.02 N/m, Bruker). The force applied was maintained at 0.250 pN with a constant approach/retract speed of 20 μm/s (*Z*-range of 500 nm), at a resolution of 250 nm × 250 nm for the adhesion images. For *in vivo* stiffness measurements, triplicate suspensions of WT and ∆*sl2*∆*eag* strains (5 μl) collected at S growth phase (OD_600_ (WT) = 5.4 ± 0.1 and OD_600_ (∆*sl2*∆*eag*) = 5.2 ± 0.2) were diluted in 10 μl of MOD and then deposited on the functionalized Petri dishes. After incubation for 25 min at RT, the remaining liquid was blotted with paper, and 1 ml of MOD was added before AFM acquisition. Images were recorded with a BL-AC40TS cantilever (mean cantilever spring constant = 0.1 N/m, Olympus). A constant force of 0.5 nN was applied, a resolution of 500 nm × 500 nm was used for mechanical analysis images. The approach part of the curves was fitted using the Hertz model and considering a triangular pyramidal tip (opening angle of 35°).

### Cell Length Measurement

Images of WT and ∆*sl2*∆*eag* cells at EE growth phase were acquired in bright field with a 100x objective (Nikon CFI Apo VC, 1.4 NA, oil immersion). Bacillus length was measured using Microbe J software (Ducret et al., 2016). Mean values were calculated from 2,920 cells for WT and 5,491 cells for ∆*sl2*∆*eag*.

### Microbial Adhesion to Solvents

WT and ∆*sl2*∆*eag* cells were grown on MODG medium and collected at exponential [OD_600_ (WT) = 0.41 ± 0.1 and OD_600_ (∆*sl2*∆*eag*) = 0.47 ± 0.0] and stationary [OD_600_ (WT) = 4.6 ± 0.3 and OD_600_ (∆*sl2*∆*eag*) = 4.3 ± 0.2] growth phases. The surface affinity of cells for polar and non-polar solvents was determined as described by Park et al. (2019). Briefly, cells were washed twice and then resuspended in 0.15 M NaCl. Optical density (OD_400_ nm) of cell suspensions was adjusted to ~1 (H0). Then, 2.4 ml of bacterial suspension was added to 0.4 ml of xylene (non-polar solvent), chloroform (acidic polar solvent), or ethyl acetate (basic polar solvent). The mixtures were stirred for 1 min at 100 rpm in a Vibramax homogenizer (Dutscher) and allowed to decant for 15 min. The OD_400_ (H1) of the aqueous phase was then measured. The affinity of cells for the solvents (adhesion percentage) was calculated as follows: Adhesion (%) = (1−H1/H0) × 100. Data are presented as average values with standard deviations calculated based on three independent experiments.

### Self-Aggregation

WT and ∆*sl2*∆*eag* cell suspensions collected from overnight cultures in MODG medium were adjusted to OD_600_ ~ 1. An aliquot of culture suspension (1 ml) was placed in a spectrophotometer cuvette, and OD_600_ was monitored over 24 h static incubation (Château et al., 2022). Results are expressed as percentage of initial OD_600_. Experiments were performed in triplicate.

### Bacterial Adhesion (BioFilm Ring Test^**®**^)

WT and ∆*sl2*∆*eag* strains were grown in Brain Heart Infusion (BHI) medium at 30°C for 24 h. Cultures were adjusted to 10^6^ CFU/ml before distributing 200 μl aliquots in 96-well polystyrene plates, as previously described (Château et al., 2022). The adhesion capability of WT and ∆*sl2*∆*eag* strains was determined by measuring a biofilm index (BFI, Biofilm control, France) after 4, 8, and 24 h incubation at 30°C. The ∆BFI (BFI_control_ − BFI_sample_) was used to define the strength of bacterial adhesion: ∆BFI = 20, total adhesion; 10 < ∆BFI < 13, strong adhesion; ∆BFI < 6, weak adhesion; ∆BFI = 0, no adhesion (Sulaeman et al., 2010). Four replicates (four wells) were analyzed for each strain.

### Statistical Analysis

Experiments were performed with at least three biological replicates. Comparisons of multiple data were based on analysis of variance (ANOVA) followed by *post-hoc* analysis, e.g., two-way ANOVA followed by Bonferroni *post-hoc* analysis for SLP profiling, self-aggregation, and adhesion tests. Changes in length, affinity for solvents, and surface stiffness were evaluated using Student’s *t*-test. Statistical analyses were performed using GraphPad Prism software version 6.0 (GraphPad Software, San Diego, CA, United States). *p* ≤ 0.05 were considered significant.

## Results

### Phylogenetic Distribution of S-Layer-Forming *Bacillus cereus* Strains

**Figure 1:**
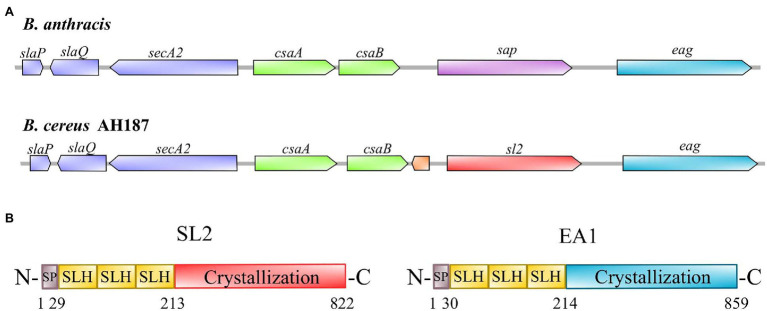
S-layer locus and primary structures of *Bacillus cereus* AH187 S-layer proteins. **(A)** S-layer locus from *Bacillus anthracis* compared to the *B. cereus* AH187 locus. The ORF located between *csaB* and *sl*2 in the *B. cereus* S-layer locus encodes an unknown protein. **(B)** Figurative representation of the primary structures of SL2 and EA1. The proteins consist of three main parts: a signal peptide (SP, gray), three S-layer homology domains (SLH, yellow), and a crystallization domain (red for SL2; blue for EA1).

In the *B. anthracis* genome, the *sap* and *eag* genes, which encode the S-layer proteins SAP and EA1, are preceded by (i) the *slaP*, *slaQ*, and *secA2* genes, which support S-layer protein secretion and, (ii) the *csaA* and *csaB* genes, which encode a pyruvyl transferase involved in anchoring the S-layer to the cell surface and its associated carbohydrate transport protein, respectively (Kern et al., 2010; Nguyen-Mau et al., 2012, 2015). All these genes belong to a genetic locus known as the S-layer cluster (Missiakas and Schneewind, 2017; Figure 1A). We searched for orthologs of the *B. anthracis* S-layer cluster in the whole genome sequences of 329 *B. cereus* strains, and identified 191 S-layer cluster-containing strains (Supplementary Table S2). Among these 191 strains, 139 belonged to mesophilic phylogroup III, mainly affiliated with subgroups III-5 (19 *B. cereus* emetic strains) or III-8 (103 *B. anthracis* strains), according to the classification described by Guinebretière et al. (Guinebretière et al., 2008). These data confirm that S-layer-forming strains are not widely dispersed (Mignot et al., 2001), and that the S-layer is a common feature of mesophilic *B. cereus* strains presenting risks for human health, such as the *B. cereus* AH187 emetic strain.

### Molecular Characteristics of *Bacillus cereus* AH187 S-Layer Proteins, SL2 and EA1

The genetic organization of the *B. cereus* AH187 S-layer cluster is illustrated in Figure 1A. BCAH187_A1064 encodes an 822-amino acid protein (B7HXP4) with a calculated molecular mass of 87.5 kDa and pI of 5.71; BCAH187_A1065 encodes an 859-amino acid protein (B7HXP5) with a calculated molecular mass of 91.0 kDa and pI of 6.41. Like their *B. anthracis* orthologs (SAP and EA1), B7HXP4 and B7HXP5 display the typical characteristics of S-layer proteins, i.e., a cleavable N-terminal signal peptide, followed by three tandem repeats of the SLH domain that bind non-covalently to SCWPs, and a C-terminal crystallization domain allowing self-assembly of the S-layer proteins (Figure 1B). The C-terminal crystallization domain of B7HXP4 is different from that of *B. anthracis* SAP, with the two proteins sharing relatively low (33%) sequence similarity (Supplementary Figure S1). In contrast, the C-terminal crystallization domain of B7HXP5 and *B. anthracis* EA1 are similar, with 89% sequence similarity. Based on these characteristics, we renamed B7HXP4 and B7HXP5, SL2 (for S-layer 2 protein) and EA1, respectively, and their genes *sl2* and *eag*.

### Morphological Features of WT and **∆***sl2***∆***eag* Mutant Cells

**Figure 2:**
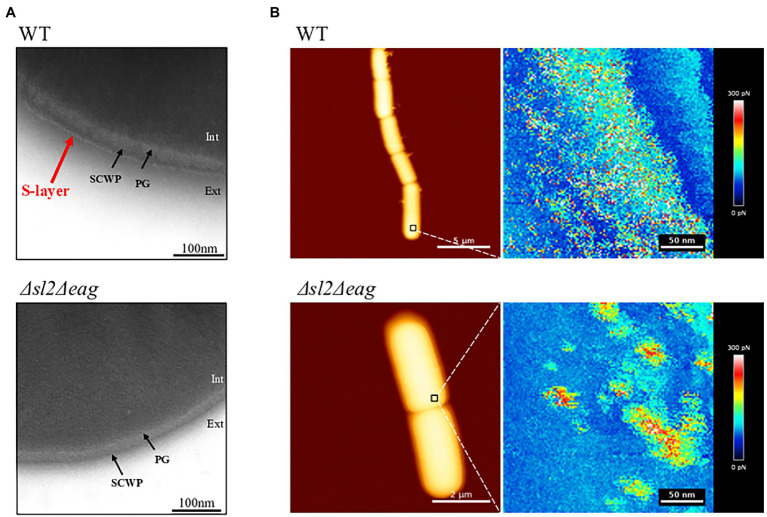
Surface structures of the WT and Δ*sl2*Δ*eag* mutant cells. **(A)** Negative-stain transmission electron microscopy (TEM) images. SCWP, Secondary Cell wall Polysaccharide; PG, peptidoglycan. **(B)** Atomic force microscopy (AFM) images showing cells immobilized on PEI-coated coverslips, and the corresponding adhesion force mapping. The colored scale of each pixel represents the intensity of the adhesion force at that location of the map.

We produced a mutant strain lacking both the *sl2* and *eag* genes by allelic replacement with a spectinomycin resistance cassette. The resulting strain was viable. Western blotting confirmed the absence of SL2 and EA1 in non-covalently attached surface protein extracts from this ∆*sl2*∆*eag* mutant strain (Supplementary Figure S2). Transmission electron microscopy (TEM) images showed that an 8 nm thick S-layer, which surrounds SCWPs on WT cells was undetectable in the ∆*sl2*∆*eag* mutant strain (Figure 2A). High-resolution AFM adhesion images confirmed a distinct surface organization in the ∆*sl2*∆*eag* mutant compared to WT (Figure 2B). Measurement of bacilli indicated that ∆*sl2*∆*eag* cells were significantly shorter than WT cells: 3.085 ± 0.010 μm compared to 3.277 ± 0.016 μm, *p* < 0.05 according to *t*-test (Supplementary Figure S3).

Taken together, these data indicate that SL2 and EA1 are required for S-layer assembly in *B. cereus* WT, and that the S-layer contributes to the surface ultrastructure and length of *B. cereus* cells.

### Dynamics of the Surface-Layer-Associated Proteome in **∆***sl2***∆***eag* Mutant and WT Cells

**Figure 3:**
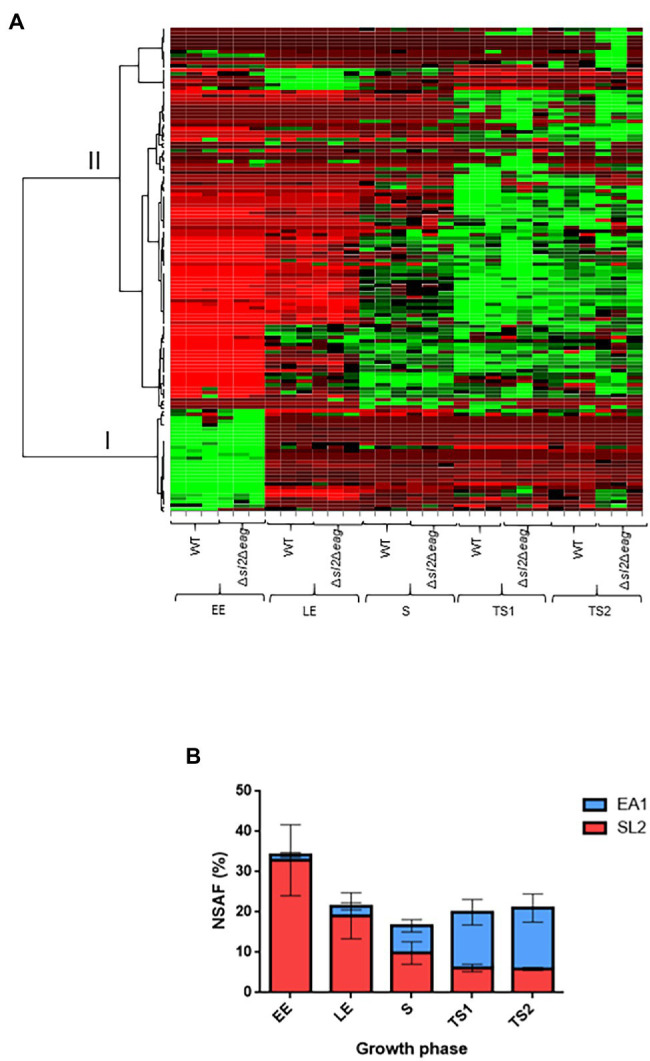
Surface-associated proteome. **(A)** Heat map representation of the surface-associated proteome in WT and Δ*sl2*Δ*eag* mutant cells. Growth phases for three biological replicates are shown at the bottom of the heat map. Protein abundances were centered and reduced before clustering. Euclidian hierarchical clustering identified two main groups: group I includes SL2 and 18 surface-layer-associated proteins (SLAP), and group II includes EA1 and other surface-associated proteins. Green and red colors indicate high and low abundance relative to the mean abundance for all proteins. **(B)** Temporal-dynamics of SL2 (red) and EA1 (blue) abundance in the surface-associated proteome for wild-type (WT). Normalized Spectral Abundance Factor (NSAF) values were used to reflect relative abundances of proteins identified in the samples. Error bars indicate the standard deviation for three biological replicates.

To study the temporal-dynamics of the surface-layer-associated proteome of *B. cereus*, we extracted non-covalently attached surface proteins from cells harvested at five time-points: EE, LE, S, TS1, and TS2 growth phases (Supplementary Figure S4). The proteomics dataset was acquired from biological triplicates for each time-point for WT and *∆sl2∆eag* mutant strains. A total of 858,556 MS/MS spectra were recorded, allowing confident identification of 1,616 proteins, based on the detection of at least two different peptides. Proteins were quantified in each sample (Supplementary Table S3). Principal component analysis (PCA) revealed good homogeneity of the proteomes of the replicates for the two strains at each time-point, and distinguished early and late exponentially growing cells from stationary cells (Supplementary Figure S5). Out of the 1,616 proteins identified, 132 were predicted to be surface-associated due to the presence of a type-I SPase-mediated N-terminal cleavage site, and/or surface anchoring domains (Supplementary Table S4). These 132 proteins, which compose the surfaceome in our conditions, included SL2 and EA1, 21 SLAPs, along with transporters, lipoproteins, proteases, and virulence factors (Supplementary Table S4). Only SL2 and EA1 were undetected in the ∆*sl2*∆*eag* mutant strain, and 13 proteins were considered differentially accumulated in extracts from the ∆*sl2*∆*eag* mutant compared to WT (value of *p* ≤ 0.05 and |log_2_ fold-change| ≥ 0.56; Supplementary Table S4). Except for one uncharacterized protein (B7HXE6), the abundance changes for these 13 proteins was growth phase-dependent. In particular, three SLAPs were detected at increased abundance in the ∆*sl2*∆*eag* mutant compared to WT at TS2 (Supplementary Table S4). Finally, lack of SL2 and EA1 did not perturb the overall *B. cereus* surfaceome profile (Figure 3A). Figure 3B shows that, in WT cells, EA1 is present at low levels at the onset of growth compared to SL2 (1.5% vs. 32% in surfaceome), and reached its maximum during the late stationary growth phase (15%) when it was 2-fold more abundant than SL2. Western blot analysis confirmed the temporal-dynamics of SL2 and EA1 expression (Supplementary Figure S6).

Based on these results, we conclude that SL2 is the main component of the *B. cereus* S-layer in growing cells, whereas EA1 is the main component of the S-layer in growth-arrested cells, and that SL2 and EA1 synthesis is growth-phase-dependent.

### Surface Distribution of EA1 and SL2 in *Bacillus cereus* AH187

**Figure 4:**
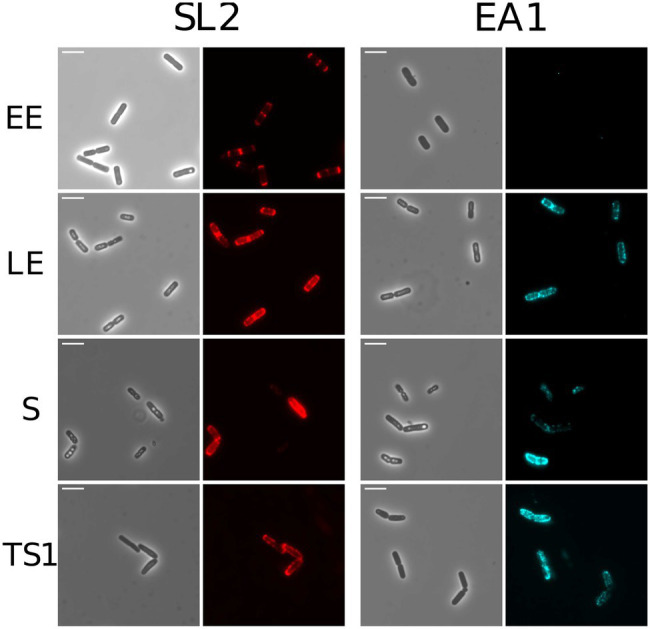
Localization of S-layer proteins in WT *B. cereus* AH187 cells. Phase contrast and fluorescence microscopy images are shown (×1000). Red: SL2; Blue: EA1. Cells shown were harvested at early exponential (EE), late exponential (LE), stationary (S), and late stationary (TS) growth phases. Scale bar, 5 μm.

Immunofluorescence microscopy was used to localize EA1 and SL2 on the bacterial cell surface. Figure 4 shows that SL2 accumulates at the cell poles in EE cells, and is more widely distributed over the cell surface at later growth stages. EA1 was undetectable at the EE growth phase, in line with its low abundance at this stage of growth. During the LE and S/TS growth phases, the fluorescence signal associated with EA1 revealed patches randomly distributed over the bacterial surface. Based on these results, we can state that SL2 and EA1 display distinct dynamic localization patterns in aerobically growing cells.

### Surface Properties of WT and S-Layer-Deficient **∆***sl2***∆***eag* Vegetative Cells

**Figure 5:**
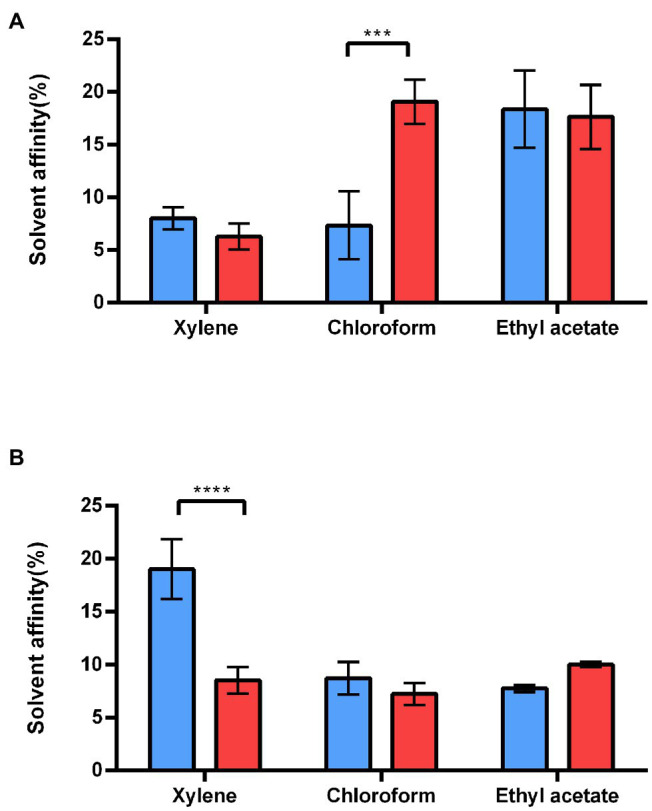
Adhesion of WT and *∆sl2∆eag* strains to solvents. **(A)** Adhesion of exponential growing WT cells (blue), and *∆sl2∆eag* cells (red) to polar (chloroform and ethyl acetate) and non-polar (xylene) solvents. **(B)** Adhesion of stationary WT cells (blue), and *∆sl2∆eag* cells (red) to polar (chloroform and ethyl acetate) and non-polar (xylene) solvents. Values correspond to mean ± SD for three biological replicates. A *t*-test was used to assess the significance of differences between the strains. ***p < 0.001; ****p < 0.0001.

To assess how the S-layer influences *B. cereus* cell surface properties, we first compared the electron-acceptor/electron-donor (Lewis acid–base) characteristics of WT and ∆*sl2*∆*eag* mutant cells (Park et al., 2019). Figure 5 shows that exponentially growing ∆*sl2*∆*eag* cells displayed a high affinity for both the basic (ethyl acetate) and acidic (chloroform) polar solvents, whereas WT cells displayed maximal affinity for the basic polar solvent (Figure 5A). No difference in affinity for polar and non-polar solvents was observed for stationary ∆*sl2*∆*eag* cells, whereas WT cells displayed maximal affinity for non-polar xylene (Figure 5B). Taken together, these data indicate that the S-layer reinforces the acidic character of bacterial cells during the exponential growth phase, and makes the cell surface less hydrophilic during the stationary phase.

**Figure 6:**
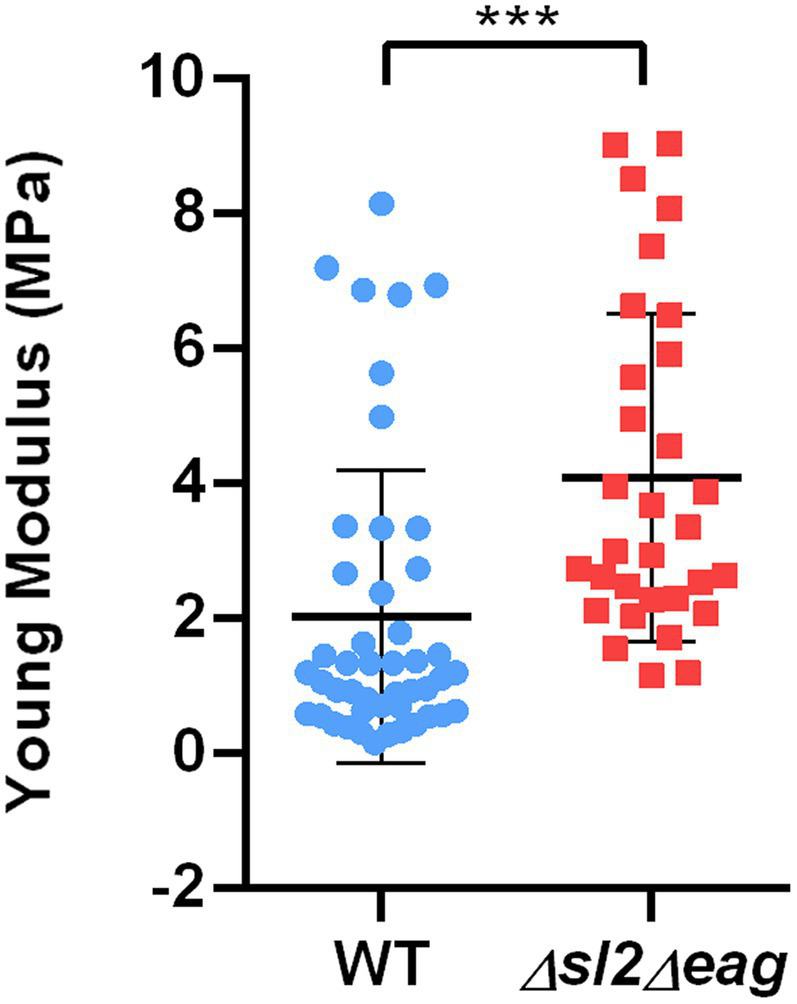
Mechanical properties of WT and *∆sl2∆eag B. cereus* strains. Young modulus (QI MOD, 500 nm x 500 nm) was measured for stationary phase cells. Boxplots were constructed using data from 46 WT cells (blue), and 31 *∆sl2∆eag* cells (red). Statistical significance was evaluated using a *t*-test. ***p < 0.001.

We then compared the stiffness of living stationary WT and ∆*sl2*∆*eag* mutant cells using AFM (Sára and Sleytr, 1987; Liu et al., 2012). Stiffness is commonly evaluated based on the Young’s modulus value (Zheng et al., 2021). Our results showed that the Young’s modulus for stationary ∆*sl2*∆*eag* cells was 2-fold higher than the Young’s modulus for WT cells (Figure 6), indicating that ∆*sl2*∆*eag* mutant cells are stiffer than WT cells at stationary growth phase. From these experiments, we concluded that the S-layer decreases the stiffness of stationary *B. cereus* cells and contributes to the mechanical properties of the *B. cereus* surface.

### Self-Aggregation and Adhesion Capacities of WT and S-Layer-Deficient **∆***sl2***∆***eag* Cells

**Figure 7:**
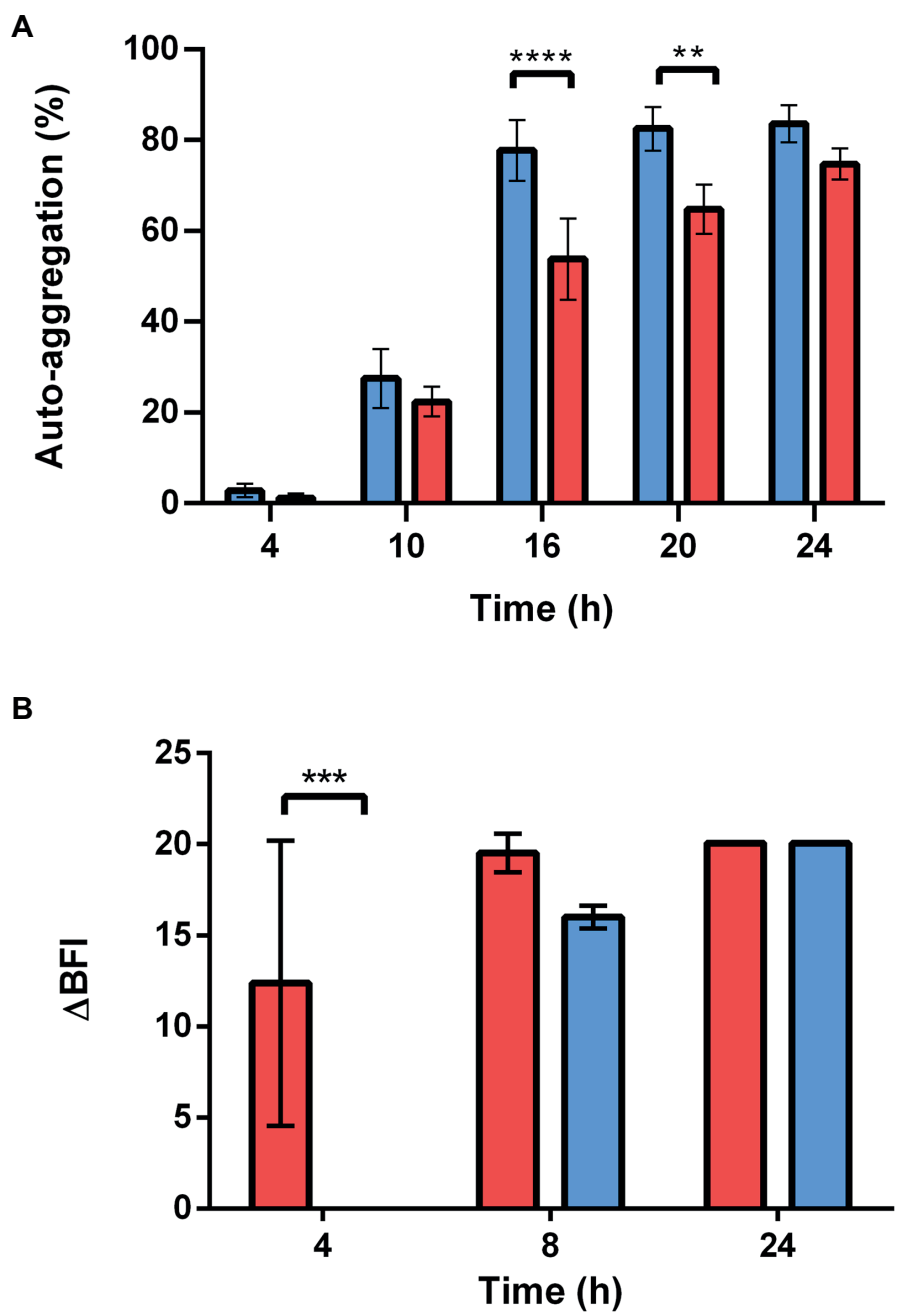
Self-aggregation and adhesion capacity of WT and *∆sl2∆eag* strains. **(A)** Self-aggregation capacity of WT and *∆sl2∆eag* suspensions collected at stationary growth phase. Values shown correspond to mean ± SD for three biological replicates. **(B)** Adhesion kinetics of WT and *∆sl2∆eag* strains at 30°C revealed by the BioFilm Ring Test®. Results are expressed as ΔBFI as a function of incubation time. ∆BFI corresponds to the biofilm index (BFI) for the control minus the BFI for the sample. Error bars correspond to the standard deviation of the mean of four biological replicates for each strain. The statistical significance of changes to self-aggregation and adhesion was analyzed based on two-way ANOVA with Bonferroni *post-hoc* tests. **p < 0.01; ***p < 0.001; ****p < 0.0001.

We evaluated the self-aggregation abilities of stationary WT and ∆*sl2*∆*eag* cells based on their sedimentation characteristics. The stationary ∆*sl2*∆*eag* cells self-aggregated more slowly than the WT cells (Figure 7A). The ability of WT and ∆*sl2*∆*eag* cells to form a biomass on a solid surface was determined using the BioFilm Ring Test® (Sulaeman et al., 2010; Château et al., 2022). The results showed that both WT and ∆*sl2*∆*eag* cells formed biofilm on microplates after 24 h of incubation (Figure 7B). However, ∆*sl2*∆*eag* cells adhered more slowly than WT cells, in line with their diminished capacity to self-aggregate.

## Discussion

Bacteria are in constant interaction with their environment. In vegetative bacteria, the cell surface is a crucial structure that mediates theses interactions. The bacterial S-layer, which is in direct contact with environment, plays a key role in the survival and persistence of bacteria (Aravindh et al., 2015; Gerbino et al., 2015). The aim of this study was to characterize the S-layer present on *B. cereus* AH187 cells. The results presented showed that the S-layer contributes to the adhesive properties of stationary *B. cereus* AH187 cells, by modulating their cell surface characteristics. The S-layer could thus enhance survival of *B. cereus* AH187 under stress conditions (Kjelleberg et al., 1982).

The S-layer of *B. cereus* AH187 is composed of two SLPs—SL2 and EA1—which are neither glycosylated nor phosphorylated (data not shown). Based on structural and genomic data, SL2 and EA1 are probably secreted at the cell surface, and anchored in the cell wall as reported for SAP and EA1 expressed by *B. anthracis* (Missiakas and Schneewind, 2017). Proteomics and microscopy analyses showed that SL2 is secreted from the early stages of growth, whereas secretion of EA1 starts later, peaking during the late stationary phase. Thus, the major protein component of the S-layer changes from SL2 during the growing phase to EA1 during the late stationary growth phase. We previously reported that synthesis of EA1 and SL2 were under the control of the master regulator of stationary phase development Spo0A (Rousset et al., 2020). However, full synthesis of SL2 could also depend on the catabolite control protein A (CcpA), which governs the overall response to carbon availability (Duport et al., 2020). Other general nutritional regulators, such as CodY, could repress the expression of S-layer genes, as described for *sap* and *eag* in *B. anthracis* (Château et al., 2013). Thus, S-layer composition probably relies on general regulatory circuits, allowing *B. cereus* to modulate the composition of its S-layer in response to nutrient conditions and developmental transitions.

The accumulation of SL2 at the cell poles, when its secretion is maximal (EE growth phase), suggests that SL2 is preferentially secreted in this region of the envelope, and thus that development of the S-layer could be coordinated at the cell poles (Janakiraman and Goldberg, 2004). No accumulation of EA1 was observed at the cell poles, rather it formed patches distributed over the cell surface, as reported for its ortholog in *B. anthracis* (Couture-Tosi et al., 2002). Due to their sequential secretion, EA1 could thus anchor to the cell wall at sites that are not already occupied by SL2.

Lack of SL2 and EA1 did not change the surface-associated proteome of *B. cereus* AH187, in particular it had no effect on the growth-phase-dependent profile of SLAPs. This result suggests that SLPs do not regulate SLAP secretion, which—like SL2 secretion—reached its maximum at the beginning of growth. Based on this accumulation profile, these proteins may share a growth-phase-dependent regulatory mechanism controlling their secretion at the cell surface.

Whereas lack of SL2 and EA1, and thus loss of the S-layer, did not affect the overall surface architecture of *B. cereus* AH187, it did slightly decrease the cell length. This morphological difference was not the result of differing growth rates between WT and ∆*sl2*∆*eag* mutant strains (Supplementary Figure S4), and thus indicates that the S-layer elongates *B. cereus* cells.

An important result from this study is that the growth-phase-dependent change to S-layer protein composition is associated with changes in the physicochemical surface properties of *B. cereus* cells. Thus, the SL2-enriched S-layer enhances the acidic character (Bellon-Fontaine, 1996) of dividing *B. cereus* cells, and the EA1-enriched S-layer decreases the hydrophilic character of stationary bacterial cells (Kotiranta et al., 1998). These data suggest that, (i) by covering dividing cells, SL2 masks the negatively charged functional groups of surface components, which include proteins, lipids, and polysaccharides, and (ii) anchoring of EA1 in the cell wall decreases the hydrophilicity of the S-layer. Thus, EA1 patches could form hydrophobic sites in the S-layer. Another possibility is that anchoring of EA1 in the cell wall induces conformational changes making the surface more hydrophobic (Xi et al., 2017). In line with this putative conformational change, we found that the S-layer decreased the surface stiffness of stationary *B. cereus* cells. This result contradicts reports for Lactobacilli (Schaer-Zammaretti and Ubbink, 2003), where the cell surfaces of S-layer-forming strains were stiffer than the surfaces of non-S-layer-forming strains. It is possible that this difference may be linked to experimental parameters, as the AFM procedure used to characterize stiffness by Schaer-Zammaretti and Ubbink differed from the procedure used here. However, it is also important to note that the S-layer composition and cell surface topography of *Lactobacilli* are quite distinct from those of *Bacilli* (Pum et al., 2013).

We showed that presence of the S-layer increased the capacity of growth-arrested *B. cereus* cells to adhere to an abiotic surface. Adhesion is governed by a number of physical, chemical, and biological parameters, including hydrophobicity, stiffness, and capacity to self-aggregate (Dufrêne, 2002; Trunk et al., 2018; Tamayo et al., 2020; Zheng et al., 2021). Here, we found that adhesion of stationary *B. cereus* cells to an abiotic surface correlated negatively with stiffness, and positively with hydrophobicity and self-aggregation capacity, all of which are mediated by the S-layer.

In conclusion, although the S-layer is not essential for *B. cereus* growth, it could contribute to stationary phase survival by enhancing the adhesive properties of growth-arrested cells. Indeed, the ability of bacterial cells to adhere to surfaces is a crucial trait for the survival of any microorganism under conditions of stress or starvation (Jaishankar and Srivastava, 2017). The benefits provided by the S-layer in these conditions are linked to the dynamics of the SLP profile, and mainly to the EA1-enrichment of the S-layer, suggesting that EA1 is a component of the bacterial stress response promoting survival in a quiescent vegetative state as an alternative to sporulation. The presence of an S-layer cluster in all emetic *B. cereus* strains, and in *B. cereus* strains classified within phylogenetic groups II, III, VI, and VII, that display some common resistance capacity toward temperature, salt, and pH (Guinebretière et al., 2008), suggests an adaptive function for the S-layer in these strains, enhancing survival in stressful environmental conditions.

## Data Availability Statement

The datasets presented in this study can be found in online repositories. The names of the repository/repositories and accession number(s) can be found in the article/Supplementary Material.

## Author Contributions

AC and CD: conceptualization. CB, AC, SL, and BA-B: methodology. CB, SL, BA-B, JA, AC, and CD: validation and writing—review and editing. CB, SL, BA-B, AC, and CD: formal analysis. CB and CD: writing—original draft preparation. All authors contributed to the article and approved the submitted version.

## Funding

This work was supported by the Platform 3A microscopy facilities, funded by the European Regional Development Fund, the French Ministry for Research, Higher Education and Innovation, the Provence-Alpes-Côte d’Azur region, the Departmental Council of Vaucluse, and the Urban Community of Avignon. The bio-AFM was funded by the REDSAIM program (Montpellier Université). CB’s PhD work was supported by a fellowship from the French Ministry for Research and Higher Education (Ministère de la Recherche et de l’Enseignement Supérieur). This work was partly supported by a grant from Avignon University.

## Conflict of Interest

The authors declare that the research was conducted in the absence of any commercial or financial relationships that could be construed as a potential conflict of interest.

## Publisher’s Note

All claims expressed in this article are solely those of the authors and do not necessarily represent those of their affiliated organizations, or those of the publisher, the editors and the reviewers. Any product that may be evaluated in this article, or claim that may be made by its manufacturer, is not guaranteed or endorsed by the publisher.
