# Supplementary figures and images for: Dynamic Profile of S-Layer Proteins Controls Surface Properties of Emetic Bacillus cereus AH187 Strain
Source: Front Microbiol. 2022 Jun 29;13:937862. doi: 10.3389/fmicb.2022.937862 (PMC9277125; doi:10.3389/fmicb.2022.937862)

**Table S1.** Oligonucleotides used in this study.


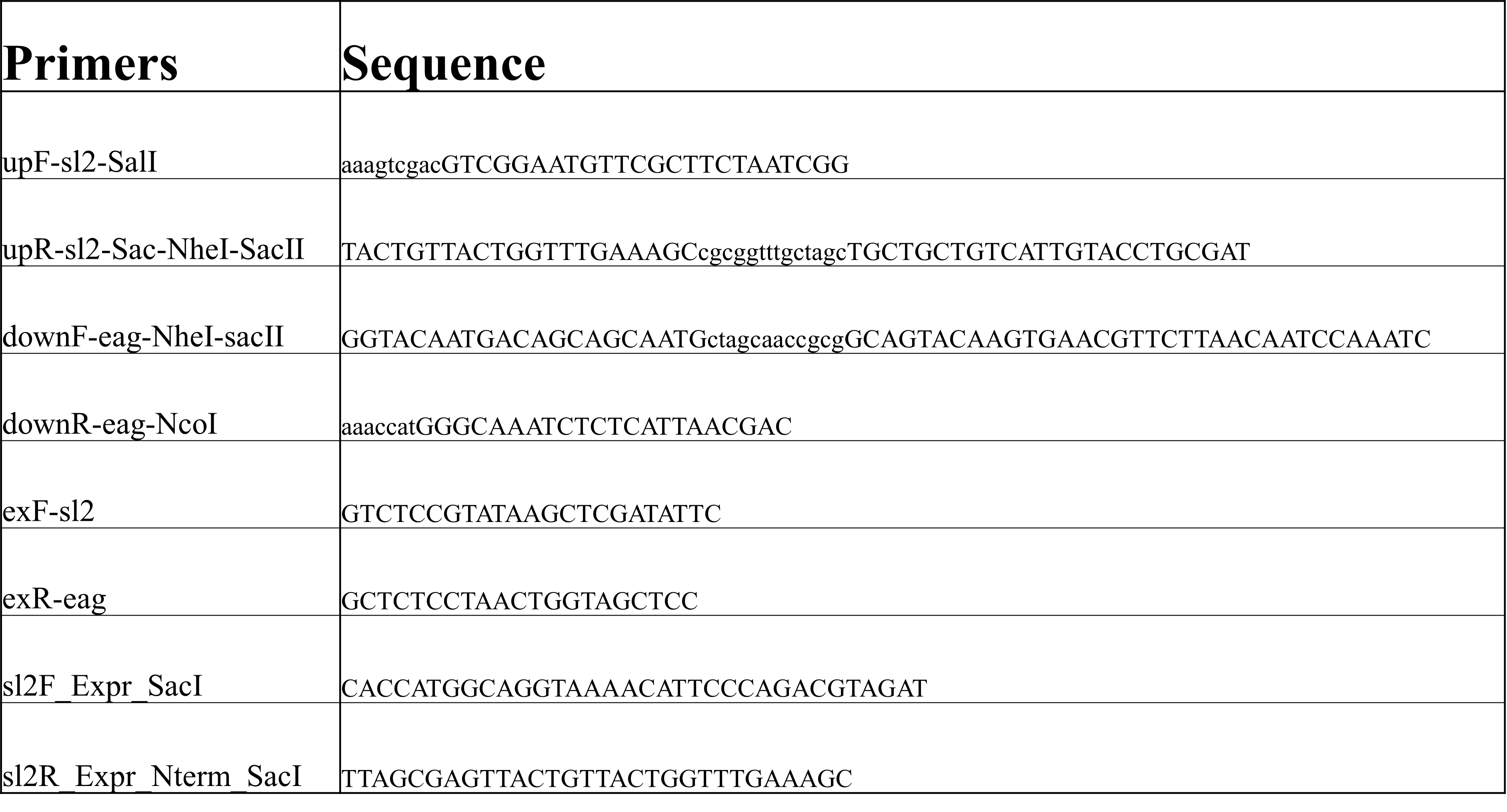

Supplement: Supplementary file 1 [file Table_1.DOCX]
