# Supplementary material for: Dynamic Profile of S-Layer Proteins Controls Surface Properties of Emetic Bacillus cereus AH187 Strain
Source: Front Microbiol. 2022 Jun 29;13:937862. doi: 10.3389/fmicb.2022.937862 (PMC9277125; doi:10.3389/fmicb.2022.937862)

**Table S2.** Distribution of S-layer cluster-containing strains within *B. cereus* phylogenetic groups*.* Analysis of 329 strains. (+) Presence and (-) Absence of the S-layer cluster.


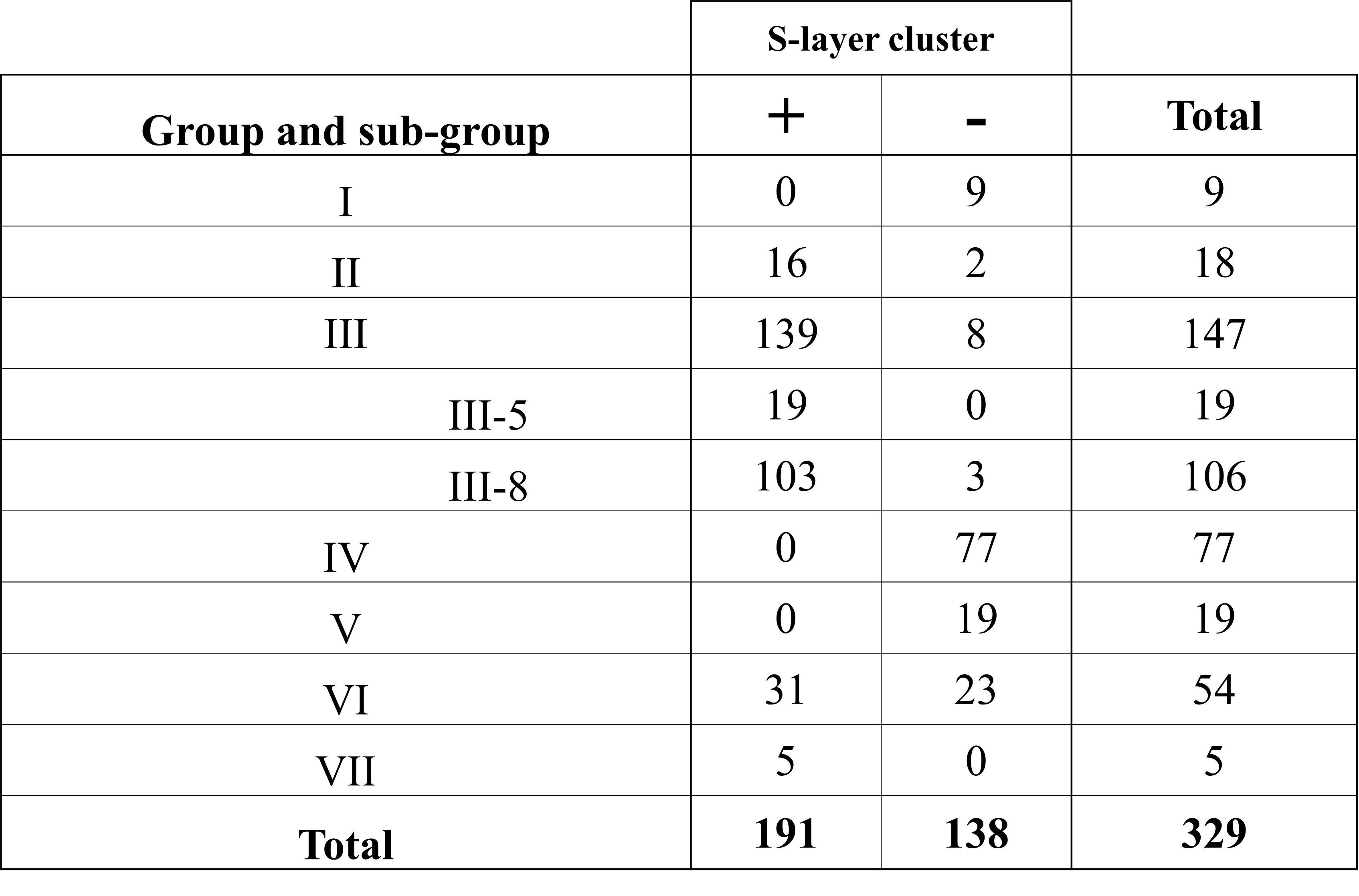

Supplement: Supplementary file 2 [file Table_2.DOCX]
