# Supplementary material for: Dynamic Profile of S-Layer Proteins Controls Surface Properties of Emetic Bacillus cereus AH187 Strain
Source: Front Microbiol. 2022 Jun 29;13:937862. doi: 10.3389/fmicb.2022.937862 (PMC9277125; doi:10.3389/fmicb.2022.937862)
Supplement: Supplementary file 5 [file Data_Sheet_1.DOCX]

**Supplementary figures**


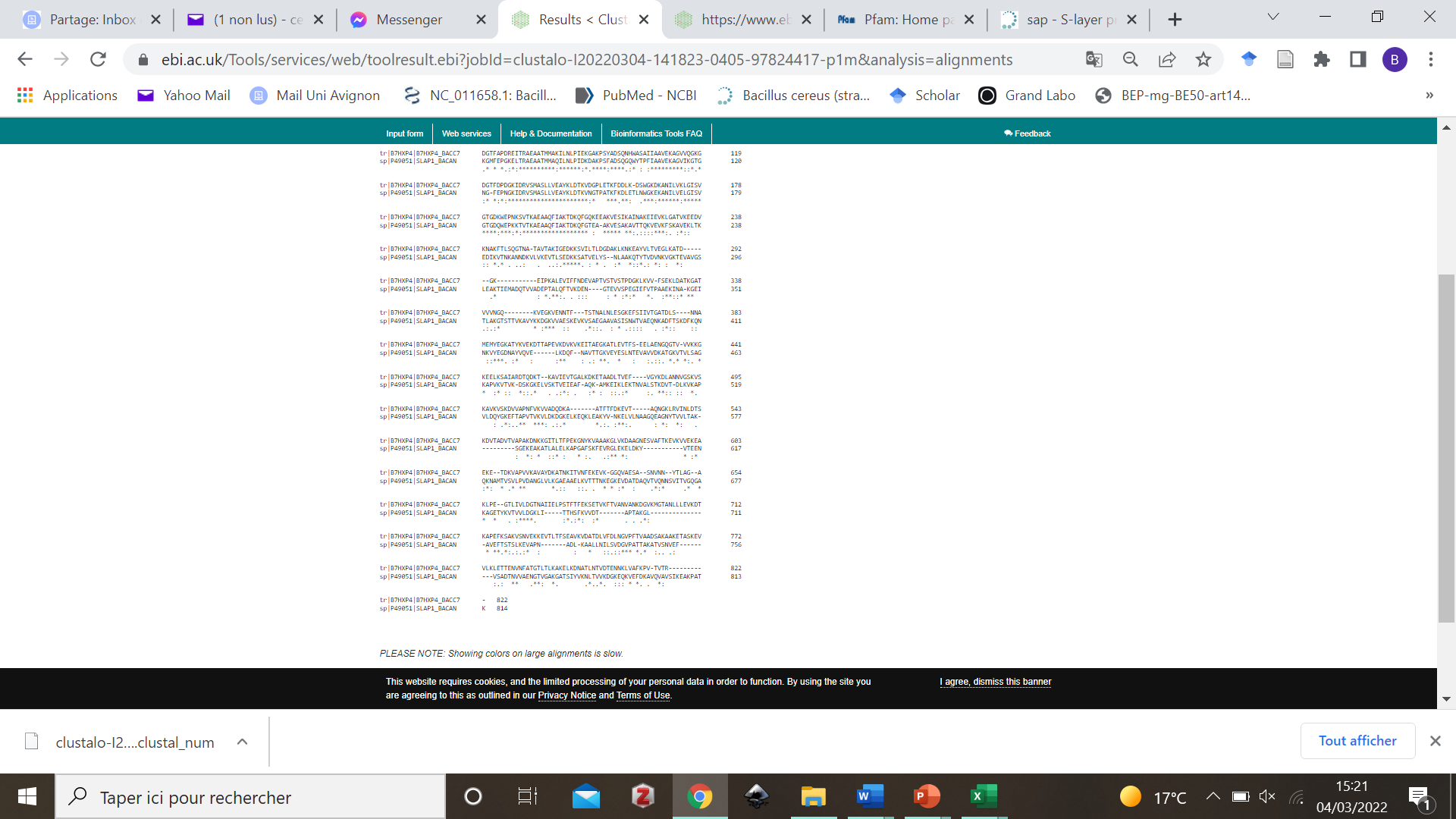


**Figure S1.** Clustal Omega sequence alignment data showing similarities and differences between *B. anthracis* SAP and *B. cereus* AH187 SL2*.*


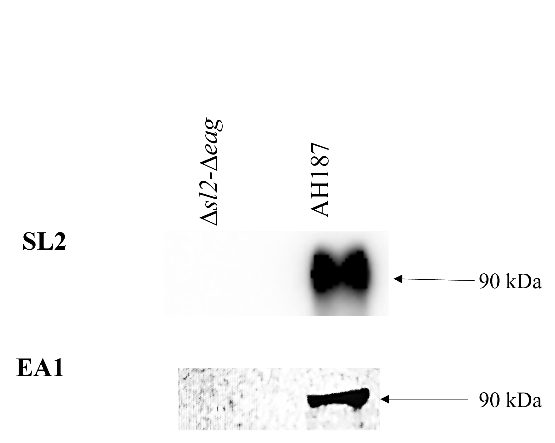


**Figure S2.** Western blot analysis of non-covalently attached surface protein extracts prepared from WT and mutant *∆sl2∆eag* cells. SL2 and EA1 were detected using rabbit polyclonal antibodies and Alexa Fluor 594-conjugated goat anti-rabbit polyclonal antibodies.





**Figure S3**. Length of WT and *∆sl2∆eag* cells measured from phase microscopy images. Boxplots were constructed from 2 920 cells for WT, and 5 491 cells for ∆*sl2*∆*eag*. A *t*-test was used to assess the significance of differences in data obtained for the two strains. ****, *P* < 0.0001.


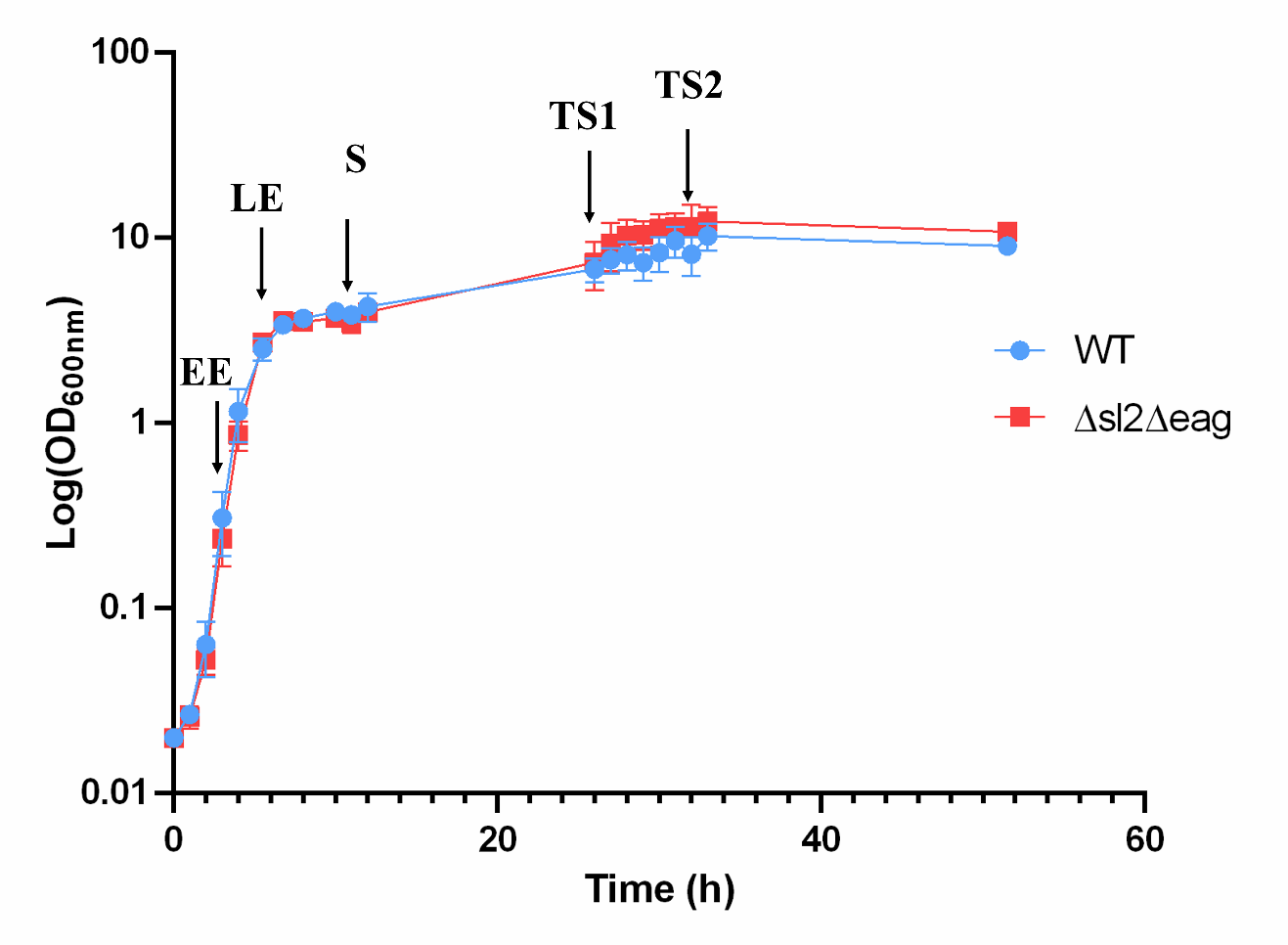


**Figure S4**. Growth curves for WT and *∆sl2∆eag* cells. Cells were grown at 37 °C in MODG medium. Error bars indicate the standard deviation for three biological replicates.

Arrows indicate sample-harvesting time-points: EE, Early exponential growth phase; LE, late exponential growth phase; S, stationary growth phase; TS1, late stationary growth phase 1; TS2, late stationary growth phase 2.


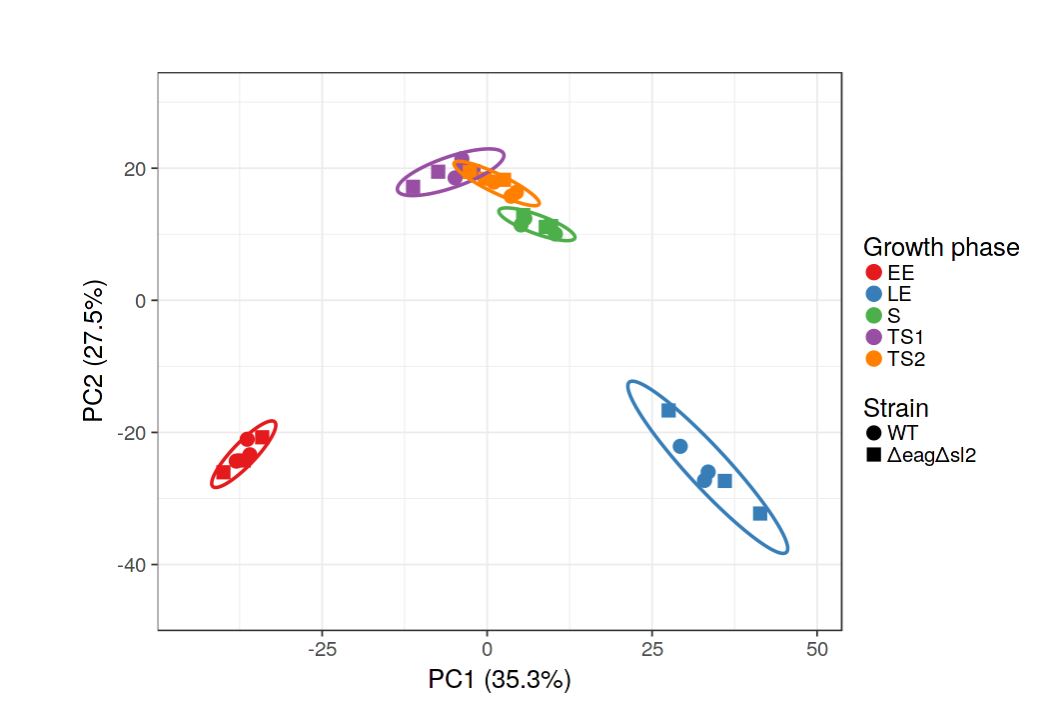


**Figure S5**. Principal component Analysis (PCA) of WT and *∆sl2∆eag* proteomics data. The PCA plot represents 1 616 proteins identified in the biological replicates for which clear proteomics profile differences between EE, LE, S, and TS growth phases were found, as indicated by colors and matching symbols. EE, Early exponential growth phase; LE, late exponential growth phase; S, stationary growth phase; TS1, late stationary growth phase 1; TS2, late stationary growth phase 2.


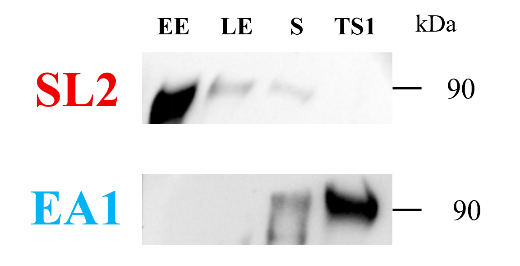


**Figure S6.** Western blot analysis of SL2 and EA1 synthesis. SL2 and EA1 were detected using rabbit polyclonal antibodies and Alexa Fluor 594-conjugated goat anti-rabbit polyclonal antibodies. EE, Early exponential growth phase; LE, late exponential growth phase; S, stationary growth phase; TS1, late stationary growth phase 1.
